# Possible Association of Multicentric Castleman's Disease with Autoimmune Lymphoproliferative Syndrome

**DOI:** 10.1089/biores.2017.0025

**Published:** 2018-04-01

**Authors:** Hiroyuki Minemura, Yoshinori Tanino, Kazuhiko Ikeda

**Affiliations:** ^1^ Department of Pulmonary Medicine, Fukushima Medical University School of Medicine, Fukushima, Japan.; ^2^ Department of Cardiology and Hematology, Fukushima Medical University School of Medicine, Fukushima, Japan.

**Keywords:** multicentric Castleman's disease, autoimmune lymphoproliferative syndrome, apoptosis, DNT cells, interleukin-6

## Abstract

Multicentric Castleman's disease (MCD) is lymphoproliferative disorder characterized by systemic inflammatory symptoms such as fever and weight loss. Human herpes virus-8 (HHV-8) is thought to be a causable pathogen in all HIV-positive and some HIV-negative MCD patients. Furthermore, the term idiopathic MCD (iMCD) was recently proposed to represent a group of HIV-negative and HHV-8-negative patients with unknown etiologies. Although the international diagnostic criteria for iMCD require exclusion of infection-related disorders, autoimmune/autoinflammatory diseases and malignant/lymphoproliferative disorders to make an iMCD diagnosis, the relationships and differences between these disorders and MCD have not yet been clarified. We recently reported the first case of MCD with autoimmune lymphoproliferative syndrome (ALPS). Although ALPS was included in the iMCD exclusion criteria as an autoimmune/autoinflammatory disease according to the international diagnostic criteria, there is a lack of evidence on the association between MCD and ALPS. In this study, we review the recent understanding of MCD and discuss the possible association between MCD with ALPS.

## Introduction

### Multicentric Castleman's disease

Dr. Benjamin Castleman first reported Castleman's disease (CD) in 1956 as unicentric lymphadenopathy with hyperplastic lymphoid tissue and hyalinized germinal centers.^1^ This type of CD is now referred to as unicentric Castleman's disease (UCD) according to the number of affected lymph nodes. Patients with UCD are asymptomatic if a solitary lymph node does not present the mass effect and are cured by complete resection. In contrast, cases of more than one affected lymph nodes with Castleman-like features were reported in HIV-positive patients after the acquired immunodeficiency syndrome was reported,^2,3^ and these cases are referred to as multicentric Castleman's disease (MCD).^4,5^ The clinical characteristics of MCD are systemic inflammatory symptoms such as fever, weight loss, night sweats, edema, ascites, pleural effusion, and splenomegaly.

In CD, three histopathological features, hyaline vascular (HV), plasma cell (PC), and mixed types, have been described. HV type is characterized by involuted germinal centers and expanded mantles formed of small lymphocytes in a laminated “onion skinning” array with penetrating hyalinized vessels. The characteristics of PC type are follicular hyperplasia with marked interfollicular plasmacytosis with hyperplastic germinal centers. The mixed type has characteristics of both HV and PC.^4,5^ The typical histopathological features of UCD and MCD are HV and PC types, respectively.

With the emergence of the HIV pandemic, human herpes virus-8 (HHV-8) was reported to be causally linked to the etiology of HIV-positive MCD.^6,7^ As a result, HHV-8 is now the well-established cause of MCD.^8^ To determine the number of affected lymph node stations, [F]-fludeoxyglucose positron emission tomography is helpful, and the intensity of [F]- fludeoxyglucose uptake has been reported to be able to distinguish MCD from lymphoma.^9^ In MCD, replication and viral-dependent hypercytokinemia are considered to be responsible for the clinicopathological findings of MCD, regardless of HIV infection.^7,8,10,11^

Interleukin (IL)-6 has been demonstrated to be expressed within the germinal centers of involved lymph nodes.^6^ HHV-8 is replicated in germinal centers, and infiltrating plasma blasts expressing viral IL-6, and induces endogenous human IL-6 in HHV-8-positive MCD.^12,13^ Some reports have shown cytogenetic abnormalities or prevalent gene polymorphisms that involve the lL-6 or lL-6 receptor genes,^14^ indicating a significant role of IL-6 in the pathogenesis of MCD. However, the etiology of MCD needs more exploration, because MCD patients with low serum IL-6 levels and who did not respond to anti-IL-6 therapy have been reported. Some other mediators, such as vascular endothelial growth factor (VEGF), IL-1, and tumor necrosis factor-α, may be involved in the pathogenesis of MCD.^9,15^

### Idiopathic MCD

MCD is a heterogeneous disorder, and there is a considerable number of MCD patients without HHV-8 or HIV infection. That there are no standard diagnostic criteria for MCD makes it difficult for us to make an accurate MCD diagnosis. To resolve this problem, the international evidence-based consensus diagnostic criteria for HHV-8-negative MCD have recently been proposed (Table 1).^16^ The term idiopathic MCD (iMCD) was proposed for HHV-8-negative patients who met these diagnostic criteria.^16^ The clinical characteristics of patients with iMCD are not different from those with MCD, and hypercytokinemia such as elevated level of IL-6 is considered to play a pivotal role in the pathogenesis of iMCD as well as MCD with specific etiologies. In iMCD patients, as well as those with MCD, although histopathological features were previously considered to be PC type, recent studies have reported 17–49% HV, 46–77% PC, and 4–20% mixed types among HIV-negative MCD cases.^9^ However, the significance of these histopathological types is not clear because transitions between HV and PC types on subsequent biopsies, as well as the simultaneous presence of both types within the same patient, have been reported. According to the international diagnostic criteria for iMCD, infection-related disorders, autoimmune/autoinflammatory diseases, and malignant/lymphoproliferative disorders must be excluded when diagnosing iMCD. However, the relationship and/or difference between these disorders and MCD have not yet been clarified. For example, because almost all enlarged lymph nodes of rheumatoid arthritis (RA) have been reported to present histopathological findings which are consistent with MCD, MCD-like features in patients with RA must be RA-related.^9^ However, because the associations between MCD and all related disorders that are mentioned in the exclusion criteria have not been clarified, further studies are necessary to compare iMCD against related disorders in detail.

**Table 1.** **Consensus Diagnostic Criteria for Idiopathic Multicentric Castleman's Disease**

I. Major criteria (need both):
1. Histopathologic lymph node features consistent with the iMCD spectrum. Features along the iMCD spectrum include (need grade 2–3 for either regressive GCs or plasmacytosis at minimum):
Regressed/atrophic/atretic germinal centers, often with expanded mantle zones composed of concentric rings of lymphocytes in an “onion skinning” appearance
FDC prominence
Vascularity, often with prominent endothelium in the interfollicular space and vessels penetrating into the GCs with a “lollipop” appearance
Sheetlike, polytypic plasmacytosis in the interfollicular space
Hyperplastic GCs
2. Enlarged lymph nodes (≥1 cm in short-axis diameter) in ≥2 lymph node stations
II. Minor criteria (need at least 2 of 11 criteria with at least 1 laboratory criterion)
Laboratory^a^
1. Elevated CRP (>10 mg/L) or ESR (>15 mm/h)^b^
2. Anemia (hemoglobin <12.5 g/dL for males, hemoglobin <11.5 g/dL for females)
3. Thrombocytopenia (platelet count <150 k/mL) or thrombocytosis (platelet count >400 k/mL)
4. Hypoalbuminemia (albumin <3.5 g/dL)
5. Renal dysfunction (eGFR <60 mL/min/1.73 m^2^) or proteinuria (total protein 150 mg/24 h or 10 mg/100 mL)
6. Polyclonal hypergammaglobulinemia (total γ globulin or immunoglobulin G > 1,700 mg/dL)
Clinical
1. Constitutional symptoms: night sweats, fever (>38°C), weight loss, or fatigue (≥2 CTCAE lymphoma score for B-symptoms)
2. Large spleen and/or liver
3. Fluid accumulation: edema, anasarca, ascites, or pleural effusion
4. Eruptive cherry hemangiomatosis or violaceous papules
5. Lymphocytic interstitial pneumonitis
III. Exclusion criteria (must rule out each of these diseases that can mimic iMCD)
Infection-related disorders
1. HHV-8 (infection can be documented by blood polymerase chain reaction, diagnosis of HHV-8-associated MCD requires positive LANA-1 staining by IHC, which excludes iMCD)
2. Clinical EBV-lymphoproliferative disorders such as infectious mononucleosis or chronic active EBV (detectable EBV viral load not necessarily exclusionary)
3. Inflammation and adenopathy caused by other uncontrolled infections (e.g., acute or uncontrolled CMV, toxoplasmosis, HIV, active tuberculosis)
Autoimmune/autoinflammatory diseases (requires full clinical criteria, detection of autoimmune antibodies alone is not exclusionary)
1. Systemic lupus erythematosus
2. Rheumatoid arthritis
3. Adult-onset still disease
4. Juvenile idiopathic arthritis
5. Autoimmune lymphoproliferative syndrome
Malignant/lymphoproliferative disorders (these disorders must be diagnosed before or at the same time as iMCD to be exclusionary):
1. Lymphoma (Hodgkin and non-Hodgkin)
2. Multiple myeloma
3. Primary lymph node plasmacytoma
4. FDC sarcoma
5. POEMS syndrome^c^
Select additional features supportive of, but not required for diagnosis
Elevated IL-6, sIL-2R, VEGF, IgA, IgE, LDH, and/or B2M
Reticulin fibrosis of bone marrow (particularly in patients with TAFRO syndrome)
Diagnosis of disorders that have been associated with iMCD: paraneoplastic pemphigus, bronchiolitis obliterans organizing pneumonia, autoimmune cytopenias, polyneuropathy (without diagnosing POEMS^c^), glomerular nephropathy, inflammatory myofibroblastic tumor.

^a^ We have provided laboratory cutoff thresholds as guidance, but we recognize that some laboratories have slightly different ranges. We suggest that you use the upper and lower ranges from your particular laboratory to determine if a patient meets a particular laboratory Minor Criterion.

^b^ Evaluation of CRP is mandatory and tracking CRP levels is highly recommended, but ESR will be accepted if CRP is not available.

^c^ POEMS is considered to be a disease “associated” with CD. Because the monoclonal plasma cells are believed to drive the cytokine storm, we do not consider it iMCD, but rather “POEMS-associated MCD.”

B2M, β-2-microglobulin; CMV, cytomegalovirus; CRP, C-reactive protein; CTCAE, common terminology for adverse events; EBV, Epstein-Barr virus; eGFR, estimated glomerular filtration rate; ESR, erythrocyte sedimentation rate; FDC, follicular dendritic cell; GC, germinal center; IHC, immunohistochemistry; LANA-1, latency-associated nuclear antigen; LDH, lactate dehydrogenase; MCD, multicentric Castleman's disease; POEMS, polyneuropathy, organomegaly, endocrinopathy, M-protein, and skin changes; TAFRO, thrombocytopenia, anasarca, myelofibrosis, renal dysfunction, and organomegaly; HHV-8, human herpes virus-8; iMCD, idiopathic MCD; CD, Castleman's disease; IL, interleukin; VEGF, vascular endothelial growth factor.

### Autoimmune lymphoproliferative syndrome

Autoimmune lymphoproliferative syndrome (ALPS) is a rare nonmalignant lymphoproliferative disorder associated with increased CD3+TCRαβ+CD4-CD8- double-negative T (DNT) cells and disorder of impaired lymphocyte apoptosis associated with defects in the FAS signaling cascade.^17,18^ The diagnostic criteria for ALPS were created by consensus in 1999 by investigators at the National Institutes of Health and revised in 2010 by a group of international investigators. Table 2 is a summary of the latest diagnostic criteria for ALPS.^19^ The clinical presentations of ALPS with lymphadenopathy, splenomegaly, and autoimmune cytopenias are caused by unregulated lymphocyte proliferation due to impaired T cell apoptosis.^17^ Increase in DNT cells and a functional defect of T cells by an *in vitro* FAS-induced apoptosis test are necessary to diagnose ALPS.^20^ In addition, an increase in DNT cells is a characteristic feature of ALPS. Lymphocyte apoptosis plays an important role for sustaining lymphocyte homeostasis, peripheral immune tolerance, and preventing autoimmunity and is triggered by activation of the cell surface receptor Fas (CD95/APO-1).^21^

**Table 2.** **Diagnostic Criteria for Autoimmune Lymphoproliferative Syndrome**

Required
1. Chronic (>6 months), nonmalignant, noninfectious lymphadenopathy or splenomegaly, or both
2. Elevated CD3+TCRαβ+CD4−CD8− DNT cells (≥1.5% of total lymphocytes or 2.5% of CD3+ lymphocytes) in the setting of normal or elevated lymphocyte counts
Accessory
Primary
1. Defective lymphocyte apoptosis (in two separate assays)
2. Somatic or germline pathogenic mutation in *FAS*, *FASLG*, or *CASP10*
Secondary
1. Elevated plasma sFASL levels (>200 pg/mL) OR elevated plasma interleukin-10 levels (>20 pg/mL) OR elevated serum or plasma vitamin B12 levels (>1,500 ng/L) OR elevated plasma interleukin-18 levels >500 pg/mL
2. Typical immunohistological findings as reviewed by an experienced hematopathologist
3. Autoimmune cytopenias (hemolytic anemia, thrombocytopenia, or neutropenia) AND elevated immunoglobulin G levels (polyclonal hypergammaglobulinemia)
4. Family history of a nonmalignant/noninfectious lymphoproliferation with or without autoimmunity

A definitive diagnosis is based on the presence of both required criteria plus one primary accessory criterion. A probable diagnosis is based on the presence of both required criteria plus one secondary accessory criterion.

*CASP10*, caspase 10; DNT, double-negative T; *FAS*, first apoptosis signal; *FASLG*, FAS ligand; sFASL, soluble FAS ligand.

### Our case of MCD with impaired lymphocyte apoptosis

We recently reported the first case of iMCD with impaired T cell apoptosis.^22^ The patient was a 37-year-old woman who presented with fatigue, dyspnea, and a slight fever. Chest radiographs and computed tomography revealed diffuse ground-glass opacities, small nodules in both lung fields, as well as both hilar and mediastinal lymphadenopathy. Histopathological findings of the lung and mediastinal lymph node biopsy showed infiltration of polyclonal and PCs, which were consistent with PC-type MCD. In addition, both plasma IL-6 and VEGF levels were elevated. A diagnosis of MCD was therefore made. According to the international diagnostic criteria of iMCD, both major histopathological criteria and seven minor criteria, including two clinical criteria (erythrocyte sedimentation rate; 76 mm/h, hemoglobin; 9.1 g/dL, platelet count; 637 k/mL, albumin; 2.0 g/dL, immunoglobulin G; 4,968 mg/dL, splenomegaly, and lymphocytic interstitial pneumonitis), were met. These results suggest that our patient had iMCD. On the contrary, the percentage of DNT cells (4.9% of total T cells) exceeded the criteria for ALPS, and FAS-induced apoptosis of lymphocytes was also impaired. The diagnostic criteria for ALPS require a mutation in *FAS*, *FASLG*, or *CASP10*, or lymphocyte apoptosis in two separate assays. However, no mutation was detected in the current case, and we performed just one assay to show the impairment of FAS-induced lymphocyte apoptosis. After careful evaluation of the clinicopathological findings, we determined that our patient had ALPS. In fact, it was reported that genetic defects are not undefined in some patients with ALPS.^17^ To the best of our knowledge, our case is the sole published report describing the possible relationship between ALPS and MCD.

### Multicentric Castleman's disease and ALPS

To date, the relationship between ALPS and MCD has not been clarified. However, in FAS/FAS ligand-deficient *lpr* and *gld* mice, DNT cells accumulate and lymphoproliferation with massive and systemic enlargement of lymph nodes can be found.^23^ The findings mimic those of the enlargement of lymph nodes in MCD. In addition, HHV-8 infection is reported to increase functional Bcl-2 homolog and mimic the activity of the antiapoptotic (Bcl)-2 family of proteins, which function to inhibit the release of cytochrome *c* from mitochondria, thereby blocking the initiation of apoptosis in HHV-8-related MCD.^24,25^ These results suggest that impaired lymphocyte apoptosis is related to MCD and can cause ALPS-like features. Although there are few studies discussing the contribution of apoptosis in the etiology of MCD, lymphocyte apoptosis may play a pivotal role on the pathogenesis of MCD and ALPS. However, we have to consider the association between MCD with ALPS carefully. ALPS has been included as an exclusion criterion in the recent international diagnostic criteria for iMCD, meaning that MCD-like features can be seen in patients with ALPS.^16^ Although ALPS must include a heterogeneous population because it is a syndrome, there is no report showing the association of MCS with ALPS except ours. In addition, gene mutations that are present in some ALPS patients, such as *FAS*, were not identified in our case, although an underlying certain genetic background is considered to be a possible cause of iMCD. Further studies are necessary to define the association between ALPS and MCD. In addition, DNT cell analysis should be included in the diagnostic criteria for MCD.

## Conclusion

MCD is a heterogeneous lymphoproliferative disorder whose precise etiology has not yet been clarified in detail. Further studies are necessary to understand the pathophysiology of MCD and clarify the precise association between ALPS and MCD.

## Abbreviations Used

ALPS: autoimmune lymphoproliferative syndrome
CASP10: caspase 10
CD: Castleman's disease
CMV: cytomegalovirus
CTCAE: common terminology for adverse events
DNT: double-negative T
eGFR: estimated glomerular filtration rate
FAS: first apoptosis signal
FASLG: FAS ligand
GC: germinal center
HHV-8: human herpes virus-8
HV: hyaline vascular
IHC: immunohistochemistry
IL: interleukin
iMCD: idiopathic MCD
MCD: multicentric Castleman's disease
PC: plasma cell
RA: rheumatoid arthritis
UCD: unicentric Castleman's disease
VEGF: vascular endothelial growth factor

## References

[1] Castleman B, Iverson L, Menendez VP Localized mediastinal lymphnode hyperplasia resembling thymoma. Cancer. 1956;9:822–830 13356266 10.1002/1097-0142(195607/08)9:4<822::aid-cncr2820090430>3.0.co;2-4

[2] Harris NL Hypervascular follicular hyperplasia and Kaposi's sarcoma in patients at risk for AIDS. N Engl J Med. 1984;310:462–463 6694687 10.1056/NEJM198402163100713

[3] Lachant NA, Sun NC, Leong LA, et al. Multicentric angiofollicular lymph node hyperplasia (Castleman's disease) followed by Kaposi's sarcoma in two homosexual males with the acquired immunodeficiency syndrome (AIDS). Am J Clin Pathol. 1985;83:27–33 3871303 10.1093/ajcp/83.1.27

[4] Dossier A, Meignin V, Fieschi C, et al. Human herpesvirus 8-related Castleman disease in the absence of HIV infection. Clin Infect Dis. 2013;56:833–842 23223599 10.1093/cid/cis1009

[5] Liu AY, Nabel CS, Finkelman BS, et al. Idiopathic multicentric Castleman's disease: a systematic literature review. Lancet Haematol. 2016;3:e163–e175 27063975 10.1016/S2352-3026(16)00006-5

[6] Dupin N, Fisher C, Kellam P, et al. Distribution of human herpesvirus-8 latently infected cells in Kaposi's sarcoma, multicentric Castleman's disease, and primary effusion lymphoma. Proc Natl Acad Sci U S A. 1999;96:4546–4551 10200299 10.1073/pnas.96.8.4546 PMC16369

[7] Soulier J, Grollet L, Oksenhendler E, et al. Kaposi's sarcoma-associated herpesvirus-like DNA sequences in multicentric Castleman's disease. Blood. 1995;86:1276–1280 7632932

[8] Barozzi P, Luppi M, Masini L, et al. Lymphotropic herpes virus (EBV, HHV-6, HHV-8) DNA sequences in HIV negative Castleman's disease. Clin Mol Pathol. 1996;49:232–235 10.1136/mp.49.4.m232 PMC408065 16696081

[9] Fajgenbaum DC, van Rhee F, Nabel CS HHV-8-negative, idiopathic multicentric Castleman disease: novel insights into biology, pathogenesis, and therapy. Blood. 2014;123:2924–2933 24622327 10.1182/blood-2013-12-545087

[10] Chang Y, Cesarman E, Pessin MS, et al. Identification of herpesvirus-like DNA sequences in AIDS-associated Kaposi's sarcoma. Science. 1994;266:1865–1869 7997879 10.1126/science.7997879

[11] Moore PS, Boshoff C, Weiss RA, et al. Molecular mimicry of human cytokine and cytokine response pathway genes by KSHV. Science. 1996;274:1739–1744 8939871 10.1126/science.274.5293.1739

[12] Yoshizaki K, Matsuda T, Nishimoto N, et al. Pathogenic significance of interleukin-6 (IL-6/BSF-2) in Castleman's disease. Blood. 1989;74:1360–1367 2788466

[13] Mori Y, Nishimoto N, Ohno M, et al. Human herpesvirus 8-encoded interleukin-6 homologue (viral IL-6) induces endogenous human IL-6 secretion. J Med Virol. 2000;61:332–335 10861641 10.1002/1096-9071(200007)61:3<332::aid-jmv8>3.0.co;2-3

[14] Stone K, Woods E, Szmania SM, et al. Interleukin-6 receptor polymorphism is prevalent in HIV-negative Castleman disease and is associated with increased soluble interleukin-6 receptor levels. PLoS One. 2013;8:e54610 23372742 10.1371/journal.pone.0054610 PMC3553080

[15] van Rhee F, Stone K, Szmania S, et al. Castleman disease in the 21st century: an update on diagnosis, assessment, and therapy. Clin Adv Hematol Oncol. 2010;8:486–498 20864917

[16] Fajgenbaum DC, Uldrick TS, Bagg A, et al. International, evidence-based consensus diagnostic criteria for HHV-8-negative/idiopathic multicentric Castleman disease. Blood. 2017;129:1646–1657 28087540 10.1182/blood-2016-10-746933 PMC5364342

[17] Shah S, Wu E, Rao VK, et al. Autoimmune lymphoproliferative syndrome: an update and review of the literature. Curr Allergy Asthma Rep. 2014;14:462 25086580 10.1007/s11882-014-0462-4 PMC4148697

[18] Fisher GH, Rosenberg FJ, Straus SE, et al. Dominant interfering Fas gene mutations impair apoptosis in a human autoimmune lymphoproliferative syndrome. Cell. 1995;81:935–946 7540117 10.1016/0092-8674(95)90013-6

[19] Oliveira JB, Bleesing JJ, Dianzani U, et al. Revised diagnostic criteria and classification for the autoimmune lymphoproliferative syndrome (ALPS): report from the 2009 NIH International Workshop. Blood. 2010;116:e35–e40 20538792 10.1182/blood-2010-04-280347 PMC2953894

[20] Magerus-Chatinet A, Stolzenberg MC, Loffredo MS, et al. FAS-L, IL-10, and double-negative CD4- CD8- TCR alpha/beta+ T cells are reliable markers of autoimmune lymphoproliferative syndrome (ALPS) associated with FAS loss of function. Blood. 2009;113:3027–3030 19176318 10.1182/blood-2008-09-179630

[21] Siegel RM, Chan FK, Chun HJ, et al. The multifaceted role of Fas signaling in immune cell homeostasis and autoimmunity. Nat Immunol. 2000;1:469–474 11101867 10.1038/82712

[22] Minemura H, Ikeda K, Tanino Y, et al. Multicentric Castleman's disease with impaired lymphocytic apoptosis. Allergol Int. 2015;64:112–114 25572569 10.1016/j.alit.2014.08.009

[23] Murphy E, Roths J Autoimmunity and lymphoproliferation: induction by mutant gene lpr, and acceleration by a male-associated factor in strain BXSB mice. In: Genetic Control of Autoimmune disease. Rose NR, Bigazzi PE, Warner NL, (eds). Elsevier North Holland: New York; pp. 207–19; 1978

[24] Burrer CM, Foight GW, Keating AE, et al. Selective peptide inhibitors of antiapoptotic cellular and viral Bcl-2 proteins lead to cytochrome *c* release during latent Kaposi's sarcoma-associated herpesvirus infection. Virus Res. 2016;211:86–88 26456186 10.1016/j.virusres.2015.10.007 PMC4792251

[25] Sarid R, Sato T, Bohenzky RA, et al. Kaposi's sarcoma-associated herpesvirus encodes a functional bcl-2 homologue. Nat Med. 1997;3:293–298 9055856 10.1038/nm0397-293

